# Predicting results of mycobacterial culture on sputum smear reversion after anti-tuberculous treatment: a case control study

**DOI:** 10.1186/1471-2334-10-48

**Published:** 2010-03-06

**Authors:** Chin-Chung Shu, Jann-Tay Wang, Chih-Hsin Lee, Jann-Yuan Wang, Li-Na Lee, Chong-Jen Yu

**Affiliations:** 1 Department of Traumatology, National Taiwan University Hospital, Taipei, Taiwan; 2 Department of Internal Medicine, National Taiwan University Hospital, Taipei, Taiwan; 3 Department of Internal Medicine, Buddhist Tzu Chi General Hospital, Taipei Branch, Taipei, Taiwan; 4 Department of Laboratory Medicine, National Taiwan University Hospital, Taipei, Taiwan

## Abstract

**Background:**

Little is currently known regarding sputum smear reversion (acid-fast smear becomes positive again after negative conversion) during anti-tuberculous treatment. This study aimed to evaluate its occurrence in patients with pulmonary tuberculosis (TB) and identify factors predicting results of mycobacterial culture for smear-reversion of sputum samples.

**Methods:**

The retrospective review was performed in a tertiary referral center and a local teaching hospital in Taiwan. From 2000 to 2007, patients with smear-positive culture-confirmed pulmonary TB experiencing smear reversion after 14 days of anti-tuberculous treatment were identified.

**Results:**

The 739 patients with smear-positive pulmonary TB had 74 (10%) episodes of sputum smear reversion that grew *Mycobacterium tuberculosis *in 22 (30%) (Mtb group). The remaining 52 episodes of culture-negative sputum samples were classified as the non-Mtb group. The anti-tuberculous regimen was modified after confirming smear reversion in 15 (20%). Fourteen episodes in the Mtb group and 15 in the non-Mtb group occurred during hospitalization. All were admitted to the negative-pressure rooms at the time of smear reversion. Statistical analysis showed that any TB drug resistance, smear reversion within the first two months of treatment or before culture conversion, and the absence of radiographic improvement before smear reversion were associated with the Mtb group. None of the smear reversion was due to viable *M. tuberculosis *if none of the four factors were present.

**Conclusions:**

Sputum smear reversion develops in 10% of patients with smear-positive pulmonary TB, with 30% due to viable *M. tuberculosis *bacilli. Isolation and regimen modification may not be necessary for all drug-susceptible patients who already have radiographic improvement and develop smear reversion after two months of treatment or after sputum culture conversion.

## Background

Tuberculosis (TB) remains a global health problem despite near eradication in some developed countries [1-4]. In 2005, the incidence was 76 per 100,000 population in Taiwan, 80 per 100,000 in the Republic of Korea, and 600 per 100000 in South Africa [1,2]. To prevent further dissemination of *Mycobacterium tuberculosis *from TB patients, adequate anti-tuberculous treatment with the implementation of the Directly Observed Therapy (DOT) is important [5]. This is meant to achieve negative conversion of sputum smear for acid-fast bacilli (AFB) and culture for *M. tuberculosis*.

In some TB patients, follow-up sputum smears occasionally reveal AFB after negative conversion (smear reversion). To avoid transmission, those with sputum smear reversion are usually admitted in a negative-pressure isolation room if hospitalization is indicated. The anti-tuberculous regimen is modified under the impression of treatment failure, drug resistance, or poor adherence. Indeed, only those with viable *M. tuberculosis *bacilli should receive proper isolation and further work-up. However, mycobacterial culture results are available only after 1-2 weeks using fluorometric culture technique, and 4-6 weeks by conventional solid culture medium [6]. The commercialized nucleic acid amplification tests, despite good sensitivity and specificity, are expensive and do not discriminate between viable and dead bacilli [7].

Therefore, from a practical standpoint, it is very important to understand how to differentiate among viable *M. tuberculosis*, dead bacilli, and non-tuberculous mycobacteria (NTM) in order to identify cases that remain infectious and reduce unnecessary expenditure of medical resources. This issue is not properly addressed in current literature. This retrospective study aimed to identify TB patients with smear reversion during anti-tuberculous treatment and to compare the clinical characteristics between those whose sputum samples remained culture-positive for *M. tuberculosis *and those whose sputum samples were culture-negative. The study also tried to identify factors predicting the results of mycobacterial culture upon sputum smear reversion after anti-tuberculosis treatment.

## Methods

### Study subjects

This study was conducted in a 2000-bed tertiary-care referral center in northern Taiwan and its branch, a 500-bed local teaching hospital in southern Taiwan. The Institutional Review Board of the hospital's Research Ethics Committee approved the protocol. By review of the database of mycobacterial laboratories, all patients with smear-positive, culture-confirmed pulmonary TB from 2000 to 2007 in the tertiary-care referral center and from 2004 to 2007 in the local teaching hospital were identified.

Patients whose sputum samples became smear-positive for AFB after having three consecutive smear-negative sputum samples and having received anti-tuberculous treatment for more than 14 days (sputum smear reversion) were identified. They were classified into the *M. tuberculosis *group (Mtb group) if their sputum samples at smear reversion were culture-positive for *M. tuberculosis*, and the non-Mtb group if their sputum samples at smear reversion were culture-negative.

### Mycobacterial study

Sputum samples were processed and pre-treated as previously described [8]. AFB smears of the processed samples were stained by the Kinyoun method and examined using standard procedures [9]. Smear grading was noted according to the American Thoracic Society guidelines [10]. If several sputum samples collected in the same period from a patient were smear-positive but with different grades, the highest was recorded as the patient's smear grading. From November 2006, the auramine-rhodamine fluorochrome method was used for screening before the smear was confirmed by the Kinyoun method [9].

The medium for primary mycobacterial isolation was Middlebrook 7H11 selective agar with antimicrobials (Remel Inc., Lexena, Kans.) and the fluorometric BACTEC technique (BACTEC Mycobacterium Growth Indicator Tube [MGIT] 960 system, Becton-Dickinson). Mycobacterial species were identified using conventional biochemical testing [9]. The susceptibility test of first-line anti-tuberculous drugs was examined using two concentrations of isoniazid (0.2 and 1.0 μg/ml) and ethambutol (7.5 and 15 μg/ml), and one concentration of rifampicin (1.0 μg/ml) [11]. For isoniazid and ethambutol, low-level resistance was defined as resistance.

### Terminology definition and data collection

The patients' medical charts and interview records from TB case managers were reviewed to collect clinical data, including age, gender, underlying co-morbidity, symptoms, and results of laboratory tests and radiographic findings upon initial diagnosis and on sputum smear reversion. Management with smear reversion and outcome at the end of follow-up were also recorded.

In the two hospitals, patients with smear-positive pulmonary TB were requested to submit three sputum samples for acid-fast smear and mycobacterial culture every two weeks until smear conversion, and then monthly until culture conversion. Sputum culture conversion after anti-tuberculous treatment was achieved if three consecutive sputum samples were culture-negative for *M. tuberculosis*. The sputum mycobacterial load during smear reversion was considered "high" if there were multiple 3+ or 4+ positive smears, "medium" if there were multiple 2+ positive smears, and "low" for the rest.

A single pulmonologist and a single radiologist both blinded to the clinical data interpreted the findings on chest radiography. If their opinions differed, the films were reviewed by another senior pulmonologist blinded to the results. Radiographically, "no improvement" was considered if the lesions seen on smear reversion did not decrease in size as compared to those before anti-tuberculous treatment, or if there were new lesions.

Standard anti-tuberculous treatment was defined as daily isoniazid, rifampicin, ethambutol, and pyrazinamide (HREZ) for the first 2 months (intensive phase), followed by daily isoniazid and rifampicin for 4 months (continuation phase) [12]. Primary-care physicians modified the regimen based on any concomitant hepatic/renal disease, adverse events, or results of susceptibility testing. The government began the Directly Observed Therapy-short course (DOTs), which covered the entire course of anti-tuberculous treatment, in April 2006. Treatment failure was defined as the presence of culturable *M. tuberculosis *in sputum samples after 4 months of anti-tuberculous treatment [13].

### Statistical analyses

Inter-group differences were compared using the *chi*-square test or Fisher exact test for categorical variables, if appropriate. Time to smear reversion and survival were compared by the Kaplan-Meier method using the log-rank test. Variables with a significant difference (*p *< 0.05) in univariate analysis were further tested by multivariate logistic regression analysis to identify independent factors predictive of the results of mycobacterial culture on sputum smear reversion.

## Results

Seven hundred and thirty-nine (739) patients with smear-positive culture-confirmed pulmonary TB were identified. The pre-treatment *M. tuberculosis *isolate was multi-drug resistant (MDR) in 49, resistant but not MDR in 114, and all susceptible in the remaining 576. There were 68 patients with 74 (10.0%) episodes of sputum smear reversion, one with three episodes, and another four with two episodes. Mycobacterial culture of sputum samples on smear reversion yielded *M. tuberculosis *in 22 episodes, NTM in 5, and negative in the remaining 47. The former 22 (30%) was defined as the Mtb group and the rest as the non-Mtb group. The median number of sputum samples collected upon smear reversion was 3 (range: 2-6 sets). Of the 46 episodes of smear reversion that developed after November 2006, nine (20%) were due to viable *M. tuberculosis *bacilli. In the Mtb group, the pre-treatment *M. tuberculosis *isolate was MDR in five (10% of the 49 patients with MDR TB) and was resistant but not MDR in six (5.3% of the 114 patients with resistant but not MDR TB).

The clinical characteristics of the two groups were similar but more patients in the Mtb group had smear reversion within the first two months of anti-tuberculous treatment and before sputum culture conversion. More patients in the Mtb group also had no improvement in symptoms before smear reversion and were not treated under DOTs (Table 1). Four in the Mtb group developed smear reversion after nine months of anti-tuberculous treatment. About half of the patients in each group had underlying diseases, which were most commonly diabetes mellitus and malignancy (Additional file 1: Table S1). Two patients in the non-Mtb group were co-infected by the human immunodeficiency virus (HIV). The Mtb group had a higher rate of resistance against tested first-line drugs and higher mycobacterial load (medium to high) on smear reversion (Table 2). A significantly higher proportion of patients in the Mtb group had no radiographic improvement before smear reversion than those in the non-Mtb group (*p *= 0.005).

**Table 1:** Clinical characteristics of patients with smear reversion.

	Mtb group (n = 22)	Non-Mtb group (n = 52)	*p *value
Age ≥ 65 years	9 (41%)	24 (46%)	0.678
Male gender	16 (73%)	43 (83%)	0.330
*Underlying co-morbid condition	10 (48%)	30 (57%)	0.334
Timing of smear reversion after treatment			
Median (range): days	68 (16 ~ 647)	135 (43 ~ 760)	0.240^†^
Within 2 months	9 (41%)	5 (10%)	0.002
Before sputum culture conversion	17 (77%)	11 (20%)	< 0.001
*Symptoms not improved at smear reversion	13 (59%)	15 (29%)	0.014
Regimen modification before smear reversion	13 (59%)	19 (37%)	0.073
Receiving direct observed therapy	9 (41%)	41 (79%)	0.002
Mortality at the end of follow-up	7 (32%)	8 (15%)	0.118
Median survival: months	12.8	11.1	0.178^†^
Treatment failure	6 (27%)	5 (10%)	0.051

Data are no. (%), unless otherwise indicated.

*The detail of underlying co-morbid condition and symptoms were described in additional file 1: Table S1.

^†^*p *value was calculated by the Kaplan-Meier method.

**Table 2:** Laboratory and radiographic findings in patients with smear reversion.

	Mtb group (n = 22)	Non-Mtb group (n = 52)	*p *value
Mycobacterial load at smear reversion			0.007
High	3 (14%)	1 (2%)	
Medium	8 (36%)	9 (17%)	
*Resistance of pre-treatment Mtb isolate			
Any TB drug resistance	11 (50%)	9 (17%)	0.004
Multidrug resistance	5 (23%)	1 (2%)	0.009
Finding of chest film			
Bilateral involvement at initial	16 (73%)	33 (63%)	0.441
Cavitary lesion at initial	7 (32%)	12 (23%)	0.431
Not improved at smear reversion	19 (86%)	27 (52%)	0.005
Blood tests at reversion			
Leukocyte (>11000 or <4000/μL)	6 (27%)	11 (21%)	0.555
Hemoglobin (< 12 g/dL)	13 (63%)	19 (42%)	0.055
Albumin (< 3.5 g/dL)	4 (36%)	7 (50%)	0.495
Total Bilirubin (> 1.2 mg/dL)	2 (11%)	6 (16%)	0.614
Creatinine (> 1.5 mg/dL)	3 (17%)	4 (9%)	0.358

Abbreviation: Mtb, *Mycobacterium tuberculosis*; TB, tuberculosis

Data are no. (%) or mean [no. of tested patients], unless otherwise indicated.

*The details of resistance of pre-treatment isolate were described in additional file 1: Table S2.

**Figure 1:**
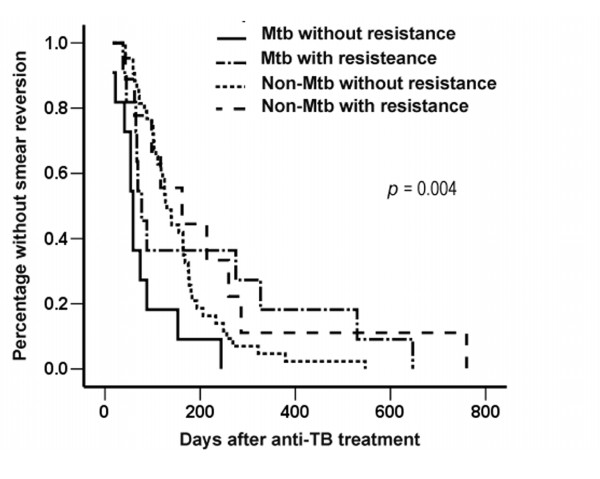
**Each of the *Mycobacterium tuberculosis *(Mtb) and non-Mtb groups was divided into two sub-groups based on susceptibility results of the pre-treatment *M. tuberculosis *isolates**. Time to smear reversion was plotted by the Kaplan Meier method and compared using log-rank test.

Both groups were divided into two sub-groups each based on the susceptibility results of the pre-treatment *M. tuberculosis *isolates, and were compared (Additional file 1: Tables S1 and S2). The findings were similar except for: 1) the proportions of patients treated under DOTs were not significantly different (*p *= 0.073); 2) the proportions of patients whose anti-tuberculous regimen had ever been modified were significantly different (*p *< 0.001); and 3) the time to smear reversion was significantly different (Figure 1).

The anti-tuberculous regimen was modified in 15 (20%) episodes after knowing smear reversion. In the 29 episodes that occurred during hospitalization, including 14 (64%) in the Mtb group and 15 (29%) in the non-Mtb group, all were isolated in a negative-pressure room on smear reversion. The survival and outcome were similar in the two groups, though the Mtb group had a slightly, but insignificantly, higher rate of treatment failure than the non-Mtb group (*p *= 0.051, Table 1).

Of the 22 episodes of *M. tuberculosis*-associated smear reversion (Additional file 1: Table S3 and S4), the anti-tuberculous regimen was modified or interrupted before smear reversion in 13 (59%). The most common causes were adverse events (seven episodes) and resistance of pre-treatment *M. tuberculosis *isolates (seven episodes). Anti-tuberculous treatment was interrupted due to ileus in one patient and poor adherence in another. In the nine episodes without treatment modification before smear reversion, four had underlying diseases while the other five occurred within the first two months of treatment. None of the patients with *M. tuberculosis*-associated smear reversion that occurred within two months of anti-tuberculous treatment had treatment failure.

Of the 22 clinical isolates of *M. tuberculosis *during smear reversion, drug susceptibility testing was repeated for 14, which revealed additional drug resistance in two isolates. Two isolates on smear reversion were multi-drug resistant strains. The pre-treatment isolates of the two patients were all-susceptible in one and rifampicin-resistant in the other.

Seven variables were included in the multivariate logistic regression analysis: treatment under DOTs strategy, presence of any TB drug resistance of the pre-treatment *M. tuberculosis *isolate, timing of smear reversion after treatment, smear reversion before sputum culture conversion, sputum mycobacterial load at reversion, and improvement of symptoms and radiographic findings before smear reversion (Additional file 2: Table S1). The results showed that factors independently predicting the results of mycobacterial culture on smear reversion were any TB drug resistance (OR: 10.33; 95% C.I.:1.47-72.60), sputum smear reversion within 2 months after anti-tuberculous treatment (OR: 16.31; 95% C.I.: 2.02-131.64), smear reversion before sputum culture conversion (OR: 29.55; 95% C.I.: 4.07-214.72), and no radiographic improvement before smear reversion (OR: 23.57; 95% C.I.: 2.49-223.38) (Table 3).

**Table 3:** Factors associated with the presence of viable *M. tuberculosis *bacilli on sputum smear reversion in multivariate analysis.

Characteristics		Episodes no.	Cul (+) for Mtb	OR (95% CI.)
Any TB drug resistance	Yes	20	11 (55%)	10.33 (1.47-72.60)
	No	54	11 (20%)	
Mycobacterial load at reversion	Medium or high	21	11 (52%)	16.74 (0.95-296.48)
	Low	53	11 (21%)	
Timing of Sm reversion after treatment	≤ 2 months	14	9 (64%)	16.31 (2.02-131.64)
	> 2 months	60	13 (22%)	
Sm reversion before Cul conversion	Yes	28	17 (61%)	29.55 (4.07-214.72)
	No	46	5 (11%)	
Findings of chest film at Sm reversion	Not improved	46	19 (41%)	23.57 (2.49-223.38)
	Improved	28	3 (11%)	

Abbreviation: Cul, culture; Mtb, *Mycobacterium tuberculosis*; Sm, smear; TB, tuberculosis

The details of multivariate analysis were listed in Additional file 2: Table S1.

In order to evaluate the impact of treatment under DOTS, this variable was forcedly included in the final model of the multivariate analysis. The results showed that DOTS was not an independent predictor of culture results (*p *= 0.435) and the ORs for the four other variables did not significantly change.

The predictive values of the four associated factors were calculated (Table 4). If three or more factors existed, the positive predictive value was 100%. If none of them was present, the negative predictive value was 100%. The positive and negative predictive values when any two factors were considered were 64% and 98%, respectively.

**Table 4:** Predicting the result of mycobacterial culture on smear reversion by four clinical factors.

Conditions	Episodes of Sm reversion	Cul (+) for Mtb	Se	Sp	PPV	NPV
All	1	1	5%	100%	100%	72%
Any three	1	1	5%	100%	100%	72%
Any two	33	21	95%	78%	64%	98%
Anyone	61	22	100%	28%	36%	100%

Abbreviation: Cul, culture; Mtb, *Mycobacterium tuberculosis*; NPV, negative predictive value; PPV, positive predictive value; Se, sensitivity; Sm, smear; Sp, specificity

The four clinical factors include: 1) any one-drug resistance; 2) smear reversion within 2 months after treatment; 3) smear reversion before culture conversion; and 4) no radiographic improvement before smear reversion

## Discussion

Sputum smear reversion after smear negative conversion during anti-tuberculous therapy is usually considered a sign of sub-optimal treatment or poor adherence [14,15]. Aside from evaluating and fostering patient compliance, new drugs are frequently added under the suspicion of emerging drug resistance. In addition, isolation in a negative-pressure room is deemed necessary if sputum smear reversion occurs during hospitalization. The results of this study suggest that sputum smear reversion develops in about 10% of patients with smear-positive, culture-confirmed pulmonary TB in an endemic area. Of these, 30% is caused by viable *M. tuberculosis*, which imply that altering treatment plans or doing contact isolation may not be necessary in the majority of those with sputum smear reversion. Therefore, understanding the factors predictive of results of mycobacterial culture upon sputum smear reversion is extremely important to reduce unnecessary medical cost.

In this study, factors independently associated with the presence of viable *M. tuberculosis *on sputum smear reversion include smear reversion within the first two months of anti-tuberculous treatment and before sputum culture conversion, the presence of drug resistance, and the absence of radiographic improvement before smear reversion. The results show that the presence of viable *M. tuberculosis *bacilli is less likely if none or one of the four factors is present. Hence, medical resources can be saved by applying the four factors to predict the results of mycobacterial culture.

The statistical analysis reveals that the absence of any radiographic improvement before sputum smear conversion is the strongest predictor for the results of mycobacterial culture. Although chest radiography is usually feasible and results can be available within the same day, the interpretation can sometimes be confounded during anti-tuberculous treatment. NTM pulmonary infection or colonization can result in higher smear grading and possibly, radiographic progression indistinguishable from pulmonary TB [16-18]. In addition, concomitant pulmonary malignancy or chronic lung disease, or paradoxical deterioration under successful treatment, renders the interpretation more difficult [19]. These factors also seriously compromise accuracy in evaluating subjective symptoms [18,20]. Nonetheless, the findings here suggest that careful interpretation and differentiation of etiologies responsible for the radiographic changes are important in managing TB patients with smear reversion.

Patients who develop *M. tuberculosis*-associated sputum smear reversion have higher any TB drug resistance rate (50%) than those whose sputum samples are culture-negative for *M. tuberculosis *on smear reversion (17%) and in the general TB population (17.7-20.4%) [11,21]. Current evidence suggests that in areas with low prevalence of drug resistance, prescription modification is not necessary even if the pre-treatment isolate is mono-drug resistant, as long as there is improvement after one month of standard treatment [22]. However, patients in the Mtb group with drug resistance had smear reversal more than 2 months so that periodic sputum examinations and imaging studies in patients with drug-resistant TB may be needed for the early detection of emerging resistance and possible treatment failure.

This study reveals that the timing of sputum smear reversion is also an important predictor for the presence of viable *M. tuberculosis *bacilli. The underlying patho-physiology is most likely the rapidly decreased viability under effective combined anti-tuberculous chemotherapy. A sub-optimal, or even failed, regimen is not an important practical concern because none of those with sputum smear reversion within two months of anti-tuberculous treatment has treatment failure. Consistent with previous studies, only smear positivity after two months of anti-tuberculous treatment is associated with poor treatment outcome [23,24].

Because sputum production is an irregular process, *M. tuberculosis *bacilli are potentially expectorated intermittently. Quantification of mycobacterial load at sputum reversion is determined by smear grading and reproducibility. However, a medium-to-high mycobacterial load on reversion reveals an insignificant association with the Mtb group by multivariate analysis. This is probably due to the presence of dead *M. tuberculosis *bacilli after anti-tuberculosis treatment. Another possible explanation may be the small sample size of the study, which limits the statistical power to detect true differences between the two groups. Underlying co-morbidity, even HIV infection, is not associated with culture-positive smear reversion under standard anti-tuberculous treatment. As shown in previous studies, HIV infection is not associated with significantly longer smear conversion time and worse prognosis [25,26].

This study has several limitations. First, although TB treatment guidelines are well established and implemented in clinical practice, the anti-tuberculous regimens and re-challenge protocols used in this study are heterogeneous due to the retrospective design. Second, the prevalence of smear reversion is likely to be underestimated because not all TB patients treated in the hospital during the study period regularly underwent sputum examinations. Third, due to the small sample size, conclusive generalizations cannot be made and other independent predictors, like drug adherence, for the presence of viable *M. tuberculosis *bacilli may not be statistically significant. Prospective, large-scale studies are warranted.

## Conclusions

Sputum smear reversion develops in 10% of patients with smear-positive, culture-confirmed pulmonary TB in an endemic area. Of these, about 30% are due to viable *M. tuberculosis *bacilli. Proper contact isolation and investigation of possible sub-optimal treatment should be prioritised in those with drug-resistant TB, those who develop smear reversion within two months after anti-tuberculous treatment or before sputum culture conversion, or those without radiographic improvement before smear reversion.

## Competing interests

All of the authors declare no financial, professional, or otherwise personal interest of any nature or kind in related product, service, and/or company.

## Authors' contributions

JY, LN, and CJ participated in designing the study. CC participated in collecting all relevant data and wrote the manuscript. JT, CH, LN and CJ were performed the data analysis. PC and JY conceived of the study, and participated in its coordination. All authors read and approved the final manuscript.

## Pre-publication history

The pre-publication history for this paper can be accessed here:

http://www.biomedcentral.com/1471-2334/10/48/prepub

## Supplementary Material

Additional file 1 **Clinical characteristics, laboratory and radiographic findings in patients with smear reversion**. Clinical characteristics, laboratory and radiographic findings of the patients with smear reversion were described, listed and compared according to the susceptibility of the patients' pre-treatment isolates. Click here for file

Additional file 2 **Factors associated with the presence of viable *M. tuberculosis *bacilli on sputum smear reversion**. A table of multivariate analysis showed the details of the association between presence of viable *M. tuberculosis *bacilli on sputum smear reversion and seven significant factors from the univariate analysis. Click here for file
