# Supplementary material for: Predicting results of mycobacterial culture on sputum smear reversion after anti-tuberculous treatment: a case control study
Source: BMC Infect Dis. 2010 Mar 6;10:48. doi: 10.1186/1471-2334-10-48 (PMC2845134; doi:10.1186/1471-2334-10-48)
Supplement: Additional file 1 — Clinical characteristics, laboratory and radiographic findings in patients with smear reversion. Clinical characteristics, laboratory and radiographic findings of the patients with smear reversion were described, listed and compared according to the susceptibility of the patients' pre-treatment isolates. [file 1471-2334-10-48-S1.DOC]

**Table S1.** Clinical characteristics of patients with smear reversion

|  | Mtb group (n=22) | |  | Non-Mtb group (n=52) | | *p* value |
| --- | --- | --- | --- | --- | --- | --- |
|  | Without resistance  (n=11) | With resistance  (n=11) |  | Without resistance  (n=43) | With resistance  (n=9) |
| Age ≥ 65 years | 3 (27%) | 6 (55%) |  | 20 (47%) | 4 (44%) | 0.606 |
| Male gender | 10 (91%) | 6 (55%) |  | 36 (84%) | 7 (78%) | 0.132 |
| Underlying co-morbid condition | 6 (55%) | 4 (36%) |  | 26 (60%) | 4 (44%) | 0.334 |
| Diabetes mellitus | 3 | 3 |  | 16 | 3 |  |
| Malignancy | 4 | 1 |  | 6 | 2 |  |
| Acquired immunodeficiency syndrome | 0 | 0 |  | 1 | 1 |  |
| Renal failure | 0 | 0 |  | 3 | 1 |  |
| Cirrhosis of liver | 0 | 0 |  | 3 | 0 |  |
| Autoimmune diseases | 1 | 0 |  | 2 | 0 |  |
| Timing of smear reversion after treatment |  |  |  |  |  |  |
| Median (range): days | 59 (16 ~ 244) | 77 (38 ~ 647) |  | 130 (43 ~ 547) | 162 (43 ~ 760) | 0.004^†^ |
| Within 2 months | 7 (64%) | 2 (18%) |  | 4 (9%) | 1 (11%) | 0.001 |
| Before sputum culture conversion | 8 (73%) | 9 (82%) |  | 9 (21%) | 2 (22%) | < 0.001 |
| Symptoms not improved at smear reversion | 5 (45%) | 8 (73%) |  | 13 (30%) | 2 (22%) | 0.047 |
| Cough | 2 | 0 |  | 3 | 1 |  |
| Fever | 1 | 1 |  | 1 | 0 |  |
| Dyspnea | 3 | 1 |  | 7 | 0 |  |
| Chest pain | 1 | 1 |  | 1 | 0 |  |
| Others* | 1 | 1 |  | 3 | 0 |  |
| Regimen modification before smear reversion | 2 (18%) | 11 (100%) |  | 16 (37%) | 3 (33%) | < 0.001 |
| Receiving direct observed therapy | 5 (45%) | 4 (36%) |  | 30 (70%) | 7 (78%) | 0.093 |
| Mortality at the end of follow-up | 3 (27%) | 4 (36%) |  | 7 (16%) | 1 (11%) | 0.412 |
| Median survival: months | 12.8 | 13.3 |  | 11.0 | 11.2 | 0.564^†^ |
| Treatment failure | 2 (18%) | 4 (36%) |  | 4 (9%) | 1 (11%) | 0.153 |

Data are no. (%), unless otherwise indicated.

*p* values represent the significance of difference among the four groups.

*Other symptoms include gastro-intestinal symptoms, conscious change, hypotension and hemoptysis.

^†^*p* value was calculated by the Kaplan-Meier method.

**Table S2.** Laboratory and radiographic findings in patients with smear reversion

|  | Mtb group (n=22) | |  | Non-Mtb group (n=52) | | *p*  value |
| --- | --- | --- | --- | --- | --- | --- |
|  | Without resistance  (n=11) | With resistance  (n=11) |  | Without resistance  (n=43) | With resistance  (n=9) |
| Mycobacterial load at smear reversion |  |  |  |  |  | 0.037 |
| High | 2 (9%) | 1 (9%) |  | 1 (2%) | 0 |  |
| Medium | 3(23%) | 5 (45%) |  | 6 (14%) | 3 (33%) |  |
| Resistance of pre-treatment Mtb isolate |  |  |  |  |  |  |
| Any TB drug resistance | - | 11 (100%) |  | - | 9 (100%) | < 0.001 |
| Isoniazid | - | 10 (91%) |  | - | 9 (100%) |  |
| Rifampicin | - | 6 (55%) |  | - | 1 (11%) |  |
| Ethambutol | - | 3 (27%) |  | - | 0 |  |
| Multidrug resistance | - | 5 (45%) |  | - | 1 (11%) | < 0.001 |
| Finding of chest film |  |  |  |  |  |  |
| Bilateral involvement at initial | 9 (73%) | 7 (64%) |  | 28 (65%) | 5 (56%) | 0.635 |
| Cavitary lesion at initial | 3 (32%) | 4 (36%) |  | 11 (25%) | 1 (11%) | 0.643 |
| Not improved at smear reversion | 10 (86%) | 9 (82%) |  | 24 (56%) | 3 (33%) | 0.022 |
| Blood tests at reversion |  |  |  |  |  |  |
| Leukocyte (>11000 or <4000 /µL) | 2 (24%) | 4 (36%) |  | 8 (19%) | 3 (33%) | 0.774 |
| Hemoglobin (< 12 g/dL) | 6 (63%) | 7 (64%) |  | 15 (35%) | 4 (44%) | 0.269 |
| Albumin (< 3.5 g/dL) | 1 (36%) | 3 (27%) |  | 6 (14%) | 1 (56%) | 0.468 |
| Total Bilirubin (> 1.2 mg/dL) | 1 (11%) | 1 (9%) |  | 5 (12%) | 1 (11%) | 0.964 |
| Creatinine (> 1.5 mg/dL) | 0 | 3 (27%) |  | 2 (5%) | 2 (22%) | 0.070 |

Abbreviation: Mtb, *Mycobacterium tuberculosis*; TB, tuberculosis

Data are no. (%).

*p* values represent the significance of difference among the four groups.

**Table S3. Details of *Mycobacterium tuberculosis*-associated smear reversion in patients with pre-treatment isolates that were resistant strain.**

| **No.** | **Age/**  **sex** | **DOT** | **Timing of smear conversion/reversion (days after treatment)** | **Mycobacterial load at smear reversion** | **Comorbid condition** | **Resistance** | **Causes for treatment interruption** | **Symptoms not improved*** | **Cavitary lesion** | **Findings of chest film*** |
| --- | --- | --- | --- | --- | --- | --- | --- | --- | --- | --- |
| 1 | 95/M | N | 56 / 647 | High |  | MDR | Resistance | Dyspnea | Absence | Stationary |
| 2 | 94/M | N | 56 / 327 | High |  | RIF | Resistance | Cough | Absence | Stationary |
| 3 | 86/F | Y | 23 / 38 | Low |  | INH |  |  | Absence | Stationary |
| 4 | 72/F | Y | 48 / 69 | Low | DM | INH | AE |  | Absence | Stationary |
| 5 | 71/M | N | 22 / 45 | Low | DM | INH, EMB | Resistance, AE | Sputum | Absence | Improved |
| 6 | 65/M | N | 278 / 530 | Medium | DM | MDR | Resistance | Cough | Presence | Progressed |
| 7 | 55/M | Y | 57 / 88 | Medium |  | INH | AE |  | Presence | Progressed |
| 8 | 53/F | N | 43 / 67 | Low | Gastric ca. | INH | Ileus | Severe nausea | Absence | Progressed |
| 9 | 43/M | N | 49 / 65 | Low |  | MDR | Resistance, AE | Dyspnea | Absence | Progressed |
| 10 | 33/F | N | 16 / 77 | Low |  | MDR | Resistance | Cough | Presence | Improved |
| 11 | 26/F | N | 10 / 275 | Low |  | MDR | Resistance, AE | Sputum | Presence | Progressed |

Abbreviations: AE, adverse event; ca, cancer; DM, diabetes mellitus; EMB, ethambutol; INH, isoniazid; MDR, multi-drug resistance; NPC, naso-pharyngeal carcinoma; RIF, rifampicin; SLE, systemic lupus erythematosus; TB, tuberculosis

*At sputum smear positive reversion

**Table S4. Details of *Mycobacterium tuberculosis*-associated smear reversion in patients with pre-treatment isolates that were all-susceptible strain.**

| **No.** | **Age/**  **sex** | **DOT** | **Timing of smear conversion/reversion (days after treatment)** | **Mycobacterial load at smear reversion** | **Comorbid condition** | **Causes for treatment interruption** | **Symptoms not improved*** | **Cavitary lesion** | **Findings of chest film*** |
| --- | --- | --- | --- | --- | --- | --- | --- | --- | --- |
| 1 | 79/M | N | 1 / 22 | Low |  |  | Dyspnea | Absence | Stationary |
| 2 | 78/M | N | 1 / 16 | Low | DM, prostate ca. |  |  | Presence | Progressed |
| 3 | 69/M | Y | 17/59 | Medium | SLE | AE | Fever | Absence | Stationary |
| 4 | 63/M | Y | 67 / 244 | Low | Lung ca. | Poor adherence |  | Presence | Progressed |
| 5 | 63/M | N | 12 / 88 | Low | NPC |  | Fever | Absence | Progressed |
| 6 | 63/M | N | 12 / 54 | Low | NPC |  | Tarry stool | Absence | Stationary |
| 7 | 62/M | N | 23 / 74 | Medium | DM | AE | Dyspnea | Absence | Stationary |
| 8 | 58/F | Y | 27 / 153 | Low |  |  |  | Absence | Stationary |
| 9 | 56/M | Y | 33 / 54 | Low |  |  |  | Absence | Stationary |
| 10 | 35/M | N | 22 / 41 | Low | DM |  |  | Presence | Improved |
| 11 | 24/M | Y | 31 / 59 | Medium |  |  |  | Absence | Stationary |

Abbreviations: AE, adverse event; ca, cancer; DM, diabetes mellitus; EMB, ethambutol; INH, isoniazid; MDR, multi-drug resistance; NPC, naso-pharyngeal carcinoma; RIF, rifampicin; SLE, systemic lupus erythematosus; TB, tuberculosis

*At sputum smear positive reversion
