# Supplementary material for: Predicting results of mycobacterial culture on sputum smear reversion after anti-tuberculous treatment: a case control study
Source: BMC Infect Dis. 2010 Mar 6;10:48. doi: 10.1186/1471-2334-10-48 (PMC2845134; doi:10.1186/1471-2334-10-48)
Supplement: Additional file 2 — Factors associated with the presence of viable M. tuberculosis bacilli on sputum smear reversion. A table of multivariate analysis showed the details of the association between presence of viable M. tuberculosis bacilli on sputum smear reversion and seven significant factors from the univariate analysis. [file 1471-2334-10-48-S2.DOC]

**Table S1.** Factors associated with the presence of viable *M. tuberculosis* bacilli on sputum smear reversion

| **Characteristics** | | **Episodes no.** | **Culture-positive for *M. tuberculosis*** | **Univariate *p* value** | **Multivariate *p* value** | **Multivariate OR (95% CI.)** |
| --- | --- | --- | --- | --- | --- | --- |
| Treatment under direct observed therapy strategy | No | 24 | 13 (54%) | 0.002 | 0.432 |  |
|  | Yes | 50 | 9 (18%) |  |  |  |
|  |  |  |  |  |  |  |
| Any TB drug resistance | Yes | 20 | 11 (55%) | 0.004 | 0.019 | 10.33 (1.47 ~ 72.60) |
|  | No | 54 | 11 (20%) |  |  |  |
|  |  |  |  |  |  |  |
| Mycobacterial load at reversion | Medium or high | 21 | 11 (52%) | 0.007 | 0.446 | 16.74 (0.95 ~ 296.48) |
|  | Low | 53 | 11 (21%) |  |  |  |
|  |  |  |  |  |  |  |
| Timing of smear reversion after treatment | ≤ 2 months | 14 | 9 (64%) | 0.002 | 0.009 | 16.31 (2.02 ~ 131.64) |
|  | > 2 months | 60 | 13 (22%) |  |  |  |
|  |  |  |  |  |  |  |
| Smear reversion before culture conversion | Yes | 28 | 17 (61%) | < 0.001 | 0.001 | 29.55 (4.07 ~ 214.72) |
|  | No | 46 | 5 (11%) |  |  |  |
|  |  |  |  |  |  |  |
| Symptoms at smear reversion | Not improved | 28 | 13 (46%) | 0.014 | 0.568 |  |
|  | Improved | 46 | 9 (20%) |  |  |  |
|  |  |  |  |  |  |  |
| Findings of chest film at smear reversion | Not improved | 46 | 19 (41%) | 0.005 | 0.006 | 23.57 (2.49 ~ 223.38) |
|  | Improved | 28 | 3 (11%) |  |  |  |

Abbreviation: TB, tuberculosis
